# Activity-dependent serotonergic excitation of callosal projection neurons in the mouse prefrontal cortex

**DOI:** 10.3389/fncir.2014.00097

**Published:** 2014-08-26

**Authors:** Emily K. Stephens, Daniel Avesar, Allan T. Gulledge

**Affiliations:** ^1^Department of Physiology and Neurobiology, Geisel School of Medicine at DartmouthLebanon, NH, USA; ^2^Program in Experimental and Molecular Medicine, Dartmouth CollegeHanover, NH, USA

**Keywords:** serotonin, 5-HT_2A_ receptor, 5-HT_1A_ receptor, prefrontal cortex, executive function, pyramidal neuron, mouse

## Abstract

Layer 5 pyramidal neurons (L5PNs) in the mouse prefrontal cortex respond to serotonin (5-HT) according to their long-distance axonal projections; 5-HT_1A_ (1A) receptors mediate inhibitory responses in corticopontine (CPn) L5PNs, while 5-HT_2A_ (2A) receptors can enhance action potential (AP) output in callosal/commissural (COM) L5PNs, either directly (in “COM-excited” neurons), or following brief 1A-mediated inhibition (in “COM-biphasic” neurons). Here we compare the impact of 5-HT on the excitability of CPn and COM L5PNs experiencing variable excitatory drive produced by current injection (DC current or simulated synaptic current) or with exogenous glutamate. 5-HT delivered at resting membrane potentials, or paired with subthreshold depolarizing input, hyperpolarized CPn and COM-biphasic L5PNs and failed to promote AP generation in COM-excited L5PNs. Conversely, when paired with suprathreshold excitatory drive generating multiple APs, 5-HT suppressed AP output in CPn L5PNs, enhanced AP generation in COM-excited L5PNs, and generated variable responses in COM-biphasic L5PNs. While COM-excited neurons failed to respond to 5-HT in the presence of a 2A receptor antagonist, 32% of CPn neurons exhibited 2A-dependent excitation following blockade of 1A receptors. The presence of pharmacologically revealed 2A receptors in CPn L5PNs was correlated with the duration of 1A-mediated inhibition, yet biphasic excitatory responses to 5-HT were never observed, even when 5-HT was paired with strong excitatory drive. Our results suggest that 2A receptors selectively amplify the output of COM L5PNs experiencing suprathreshold excitatory drive, while shaping the duration of 1A-mediated inhibition in a subset of CPn L5PNs. Activity-dependent serotonergic excitation of COM L5PNs, combined with 1A-mediated inhibition of CPn and COM-biphasic L5PNs, may facilitate executive function by focusing network activity within cortical circuits subserving the most appropriate behavioral output.

## INTRODUCTION

The prefrontal cortex (PFC) provides “top-down” executive control of behavior, and prefrontal processing is profoundly influenced by a variety of neuromodulatory transmitters, including serotonin (5-HT; for review, see Robbins and Roberts, 2007; Puig and Gulledge, 2011). Deficits in serotonergic input to the PFC impair executive control in rats (Geyer et al., 1976; Winstanley et al., 2004a; Koot et al., 2012), monkeys (Clarke et al., 2004, 2005), and humans (LeMarquand et al., 1998; Worbe et al., 2014), as typified by inappropriate response selection, impulsivity, and/or perseverative behavior. Serotonergic mechanisms in the PFC are also implicated in episodic (Bekinschtein et al., 2013) and working (Williams et al., 2002) memory.

Cortical neurons respond to 5-HT primarily through three postsynaptic receptor subtypes, metabotropic G_i/o_-coupled 5-HT_1A_ (1A) and G_q_-coupled 5-HT_2A_ (2A) receptors that are expressed in subpopulations of excitatory (pyramidal) and inhibitory (non-pyramidal) cortical neurons, and ionotropic 5-HT_3_ receptors preferentially expressed in subpopulations of non-pyramidal neurons (Morales and Bloom, 1997; Willins et al., 1997; Aznar et al., 2003; Puig et al., 2004, 2010; Santana et al., 2004; Lee et al., 2010; Weber and Andrade, 2010). In cortical pyramidal neurons, postsynaptic 1A and 2A receptors mediate opposing inhibitory and excitatory responses, respectively (for review, see Puig and Gulledge, 2011), and are directly implicated in a variety of psychiatric diseases. For instance, 1A receptor density in the human PFC is inversely correlated with anxiety (Tauscher et al., 2001), while 1A agonists have anxiolytic and antidepressant effects (Goldberg and Finnerty, 1979; Feighner and Boyer, 1989; Akimova et al., 2009; Hesselgrave and Parsey, 2013). On the other hand, excessive activation of cortical 2A receptors contributes to the etiology of schizophrenia (Geyer and Vollenweider, 2008; Benekareddy et al., 2010), and 2A receptors are preferred targets for atypical antipsychotics (Meltzer et al., 1989; González-Maeso and Sealfon, 2009) and hallucinogens (Willins and Meltzer, 1997; Vollenweider et al., 1998). However, little is known regarding how cortical 1A and 2A receptors interact to facilitate normal cognitive function.

Layer 5 pyramidal neurons (L5PNs) are a major source of output from the PFC to distal cortical and subcortical brain regions. While many cortical pyramidal neurons express both 1A and 2A receptors (Ashby et al., 1994; Amargós-Bosch et al., 2004; Béïque et al., 2004; Santana et al., 2004; Wȩdzony et al., 2008; Moreau et al., 2010), most display purely inhibitory or excitatory responses to 5-HT (Davies et al., 1987; Araneda and Andrade, 1991; Tanaka and North, 1993; Foehring et al., 2002; Zhong and Yan, 2011), although biphasic responses involving both receptor subtypes also occur (Araneda and Andrade, 1991; Puig et al., 2005). We recently revealed that, in the mouse medial prefrontal cortex (mPFC), the direction of serotonergic modulation of L5PNs is correlated with their long-distance axonal projections (Avesar and Gulledge, 2012). 5-HT, acting at 2A receptors, selectively excites callosal/commissural (COM) projection neurons that innervate the contralateral cerebral hemisphere. While most COM neurons show a purely excitatory response to 5-HT (“COM-excited” neurons), a subpopulation of COM neurons respond to 5-HT with biphasic responses in which 1A-mediated inhibition is followed by 2A-dependent excitation (“COM-biphasic” neurons). On the other hand, 5-HT generates only 1A-dependent inhibition in brainstem-projecting corticopontine (CPn) neurons. Here we have tested the interaction of 5-HT receptors in COM and CPn L5PNs experiencing variable extrinsic excitatory drive. Our results suggest that selective, activity-dependent serotonergic regulation of cortical projection neurons may facilitate executive function by focusing network activity in circuits subserving the most appropriate behavioral output.

## MATERIALS AND METHODS

### ANIMALS

Experiments involved C57BL/6J (6-to-8-week-old) male and female mice according to methods approved by the Institutional Animal Care and Use Committee of Dartmouth College.

### RETROGRADE LABELING

Red or green fluorescent beads (Retrobeads, Lumafluor Inc.) were injected unilaterally into either the prelimbic cortex (to label COM neurons) or the pons (to label CPn neurons) using age-appropriate coordinates (Paxinos and Franklin, 2004). Animals were anesthetized throughout surgeries with vaporized isoflurane (∼2%). Following craniotomy, a microsyringe was lowered into the brain region of interest, and 300–700 nL of undiluted Retrobead solution was injected over a 10 min period. Animals were allowed to recover from surgery for at least 72 h before use in electrophysiological experiments. The location of dye injection was confirmed *post hoc* in coronal sections of the mPFC or brainstem.

### SLICE PREPARATION

Following isoflurane anesthesia and decapitation, brains were quickly removed into artificial cerebral spinal fluid (ACSF) containing, in mM: 125 NaCl, 25 NaHCO_3_, 3 KCl, 1.25 NaH_2_PO_4_, 0.5 CaCl_2_, 6 MgCl_2_, and 25 glucose, saturated with 95% O_2_/5% CO_2_. Coronal brain slices (250 μm thick) of the mPFC were cut using a Leica VT 1200 slicer and stored in a chamber filled with ACSF containing 2 mM CaCl_2_ and 1 mM MgCl_2_. Slices were stored at 35°C for ∼45 min, then kept at room temperature for up to 8 h prior to use in experiments.

### ELECTROPHYSIOLOGY

Slices were transferred to a recording chamber continuously perfused with oxygenated ACSF at 35–36°C and visualized with an Olympus BX51WI microscope. Whole-cell current-clamp recordings of L5PNs were made with patch pipettes (5 – 7 MΩ) filled with, in mM, 135 K-gluconate, 2 NaCl, 2 MgCl_2_, 10 HEPES, 3 Na_2_ATP, and 0.3 NaGTP (pH 7.2 with KOH). Epifluorescence illumination (Cairn Research; 470 or 530 nm LEDs) was used to identify labeled COM or CPn neurons in the prelimbic cortex for whole-cell recording. CPn neuron somata are exclusively found in layer 5, while COM neurons reside in both layers 5 and 2/3 (Morishima and Kawaguchi, 2006). In targeting layer 5 COM neurons along the narrowing dorsal–ventral axis of the medial cortex, we targeted COM neurons in the lateral half of labeled neurons (at least 250 μm from the pia) but above layer 6, as identified by higher-density somata and the presence of “inverted” pyramidal neurons (Van Brederode and Snyder, 1992). Data were acquired with Axograph software (Axograph Company) using a BVC-700 amplifier (Dagan Corporation) and an ITC-18 digitizer (HEKA Instruments). Membrane potentials were sampled at 25 kHz, filtered at 5 kHz, and corrected for a liquid junction potential of +12 mV.

5-HT (100 μM) was dissolved in ACSF and loaded into a patch pipette placed ∼50 μm from the targeted soma. After whole-cell break-in, neurons were initially classified as 5-HT- “inhibited,” “excited,” or “biphasic” based on their response to 5-HT (delivered for 1 s at ∼10 PSI) during periods of continuous AP generation (∼6 Hz) evoked by DC current injection through the recording electrode. Neurons referred to as “COM-excited” or “COM-biphasic” were classified based on this initial response to 5-HT alone, regardless of their responsiveness to 5-HT during other manipulations (e.g., 5-HT responses generated at resting membrane potentials; RMPs). Serotonergic inhibition was quantified as the duration of AP cessation, while excitatory responses were quantified as the peak increase in instantaneous spike frequency (ISF) relative to the average baseline firing frequency. Biphasic 5-HT responses were defined as a brief inhibition lasting at least 10 times the average baseline interspike interval, followed by an increase in AP frequency of at least 1 Hz. In some experiments, 5-HT receptors were selectively blocked with 1A (WAY 100635, 30 nM; Sigma–Aldrich) and/or 2A (MDL 11939, 500 nM; Tocris Bioscience) antagonists.

Somatic current injection was used to simulate excitatory synaptic input. The synaptic current waveform was modeled in NEURON (freely available at ) using a ball and stick “pyramidal” neuron with AMPA conductances (exponential rise and decay of 0.2 and 2 ms, respectively, a 500 pS maximum conductance, and reversing at 0 mV) placed at 1 μm intervals along a 1000 μm-long spiny dendrite (as in Gulledge et al., 2012). Synaptic currents generated by activation of all synaptic inputs at randomized timings twice within the 1500-ms-long simulation were recorded with a simulated voltage clamp (-70 mV) at the soma. The resulting current waveform was loaded into Axograph and used as a template for somatic current injections. Because of intrinsic cell-to-cell variability in input resistance and excitability, for each neuron the synaptic waveform was scaled in amplitude to generate ∼7 APs during baseline trials. The simulated synaptic current was then delivered 29 times at 3 s intervals, the exception being the sixth trial which was delayed 3 s due to application of 5-HT (100 μM, 1 s).

In other experiments, exogenous glutamate (1 mM; dissolved in ACSF) was focally applied from a patch pipette positioned near the proximal apical dendrite (∼50 μm from the soma). Depending on the experiment, the duration of glutamate puffs (8 to 20 ms duration) were adjusted (in 1 ms increments) to generate “just-subthreshold” (i.e., the maximum puff duration failing to generate APs) or reliably suprathreshold responses producing one or more APs. Single applications, or bursts of 5 applications (at 200 ms intervals), were delivered twenty times at 0.05 Hz, and 5-HT (100 μM, 1 s) applied from a second patch pipette midway between the fifth and sixth trials.

### STATISTICAL ANALYSES

Data are presented as mean ± SEM. Comparisons across cell groups utilized one-way ANOVAs (with Bonferroni or Tukey-Kramer post-tests), while comparisons within groups was accomplished using 2-tailed Student’s *t*-tests (paired or unpaired), or repeated measures ANOVA with IBM SPSS Statistics (version 21). Significance was defined as *p* < 0.05.

## RESULTS

### ACTIVITY-DEPENDENT SEROTONERGIC EXCITATION

**FIGURE 1:**
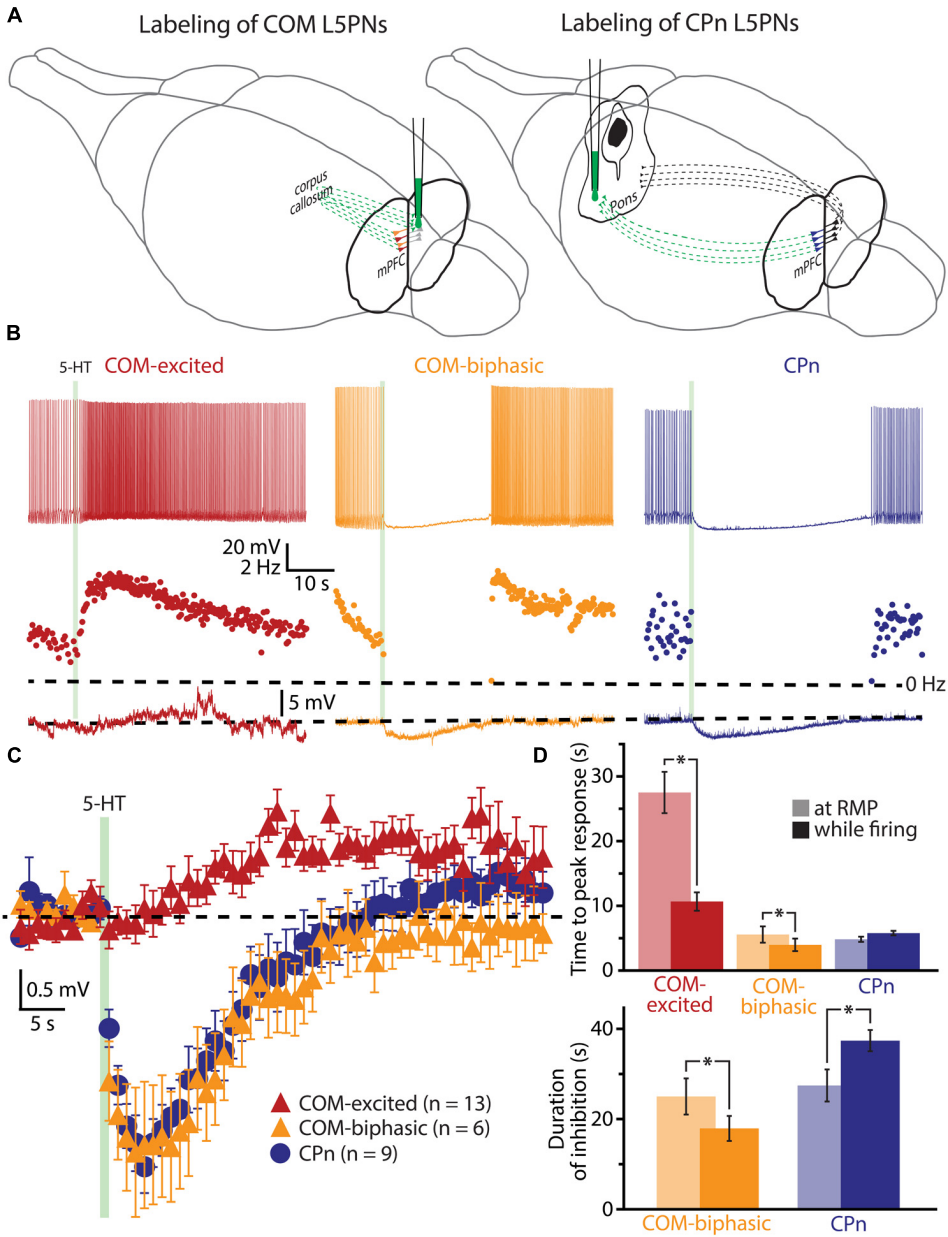
**Serotonergic excitation of COM L5PNs is more pronounced when 5-HT is paired with excitatory drive. (A)** Fluorescent Retrobeads were injected unilaterally into either the contralateral prelimbic cortex (to label COM L5PNs) or into the ipsilateral pons (to label CPn L5PNs). Dashed green lines represent the axons of cortical projection neurons conveying Retrobeads to the somata of pyramidal neurons in the mPFC. **(B)** Responses of labeled COM-excited (red), COM-biphasic (orange), or CPn (blue) neurons to focally applied 5-HT (green bar) delivered during periods of current-induced action potential (AP) generation (top) or at resting membrane potentials (RMP, bottom). Middle plots show ISF over time. Dashed-lines indicate 0 Hz (middle) or RMP (bottom). **(C)** Aggregate population responses to 5-HT application at RMPs (dashed-line) for COM-excited (red; *n* = 13), COM-biphasic (orange; *n* = 6), and CPn (blue; *n* = 9) L5PNs. Responses for each neuron were resampled at 1 Hz, and population data plotted as mean ± SEM for each resulting time point. **(D)** Comparisons of latencies to peak 5-HT responses across neuron subtypes (top) and durations of 5-HT-induced inhibition in CPn and COM-biphasic neurons (bottom), for 5-HT responses generated during periods of AP generation (opaque bars) or at RMPs (semi-opaque bars). Asterisks indicate significant differences (*p* < 0.05) between responses occurring at RMPs or while firing.

To explore the interaction of 5-HT and extrinsic depolarizing drive, we made whole-cell recordings from labeled COM and CPn L5PNs in slices of mouse mPFC. COM and CPn neurons were identified by the presence of fluorescent beads (Retrobeads) that had been injected into the contralateral prelimbic cortex, or ipsilateral pons, several days prior to slice preparation (**Figure 1A**). 5-HT (100 μM for 1 s) was delivered either at RMPs, or paired with suprathreshold DC current injection (**Figure 1B**). As previously observed (Avesar and Gulledge, 2012), 5-HT applied during current-induced activity inhibited AP generation in CPn neurons (*n* = 9), and either increased AP frequency (“excited”; *n* = 13), or generated “biphasic” (inhibitory-excitatory) responses (*n* = 6), in COM L5PNs (**Table 1**). When applied to these same neurons at RMPs, 5-HT rapidly hyperpolarized CPn (by 3.5 ± 0.4 mV) and COM-biphasic neurons (by 3.8 ± 0.7 mV), but depolarized COM-excited L5PNs by 3.3 ± 0.2 mV (**Figure 1C**). Unlike hyperpolarizing responses at RMP, serotonergic depolarization of COM-excited neurons developed slowly, with the latency to peak depolarization (28 ± 3 s) being significantly delayed relative to the latency of peak excitation for 5-HT responses occurring during DC-current-induced activity (11 ± 1 s; *n* = 13; *p* < 0.05, paired Student’s *t*-test; **Table 1** and **Figure 1D**), confirming that serotonergic excitation of COM neurons is facilitated by coincident excitatory drive (see also Araneda and Andrade, 1991; Zhang and Arsenault, 2005).

**Table 1:** Properties of L5PN subtypes and their responses to serotonin.

L5PN subtype	*n*	RMP (mV)	R_N_ (MΩ)	Sag (%)	Response property	With DC-current	At RMP
COM-excited	13	-77 ± 2	188 ± 13	6 ± 1	Peak increase in spike frequency (%) or peak depolarization (mV)	119 ± 15	3.3 ± 0.2
					Time to peak excitation (s)	11 ± 1	28 ± 3*

COM-biphasic	6	-78 ± 2	173 ± 30	4 ± 1	Peak increase in spike frequency (%)	87 ± 13	N/A
					Duration of inhibition (s)	18 ± 3	25 ± 4*
					Time to peak hyperpolarization (s)	11 ± 2	7 ± 1

CPn	9	-78 ± 1	77 ± 7	13 ± 1	Duration of inhibition (s)	37 ± 2	28 ± 4*
					Time to peak hyperpolarization (s)	6 ± 0.3	5 ± 0.4

*Asterisks indicate *p*< 0.05 vs 5-HT responses occurring during DC-evoked firing, paired Student’s *t*-test.*

The duration of inhibitory serotonergic responses in CPn and COM-biphasic neurons was also sensitive to activity state, albeit in opposite directions. In CPn neurons, the durations of spike cessations (37 ± 2 s) during current-induced depolarization were longer than were hyperpolarizations generated at RPMs (28 ± 4 s; *p* < 0.05, paired Student’s *t*-test; **Table 1**, **Figure 1D**). On the other hand, inhibitory responses in COM-biphasic neurons were longer at RMPs (25 ± 4 s) than they were during current-induced activity (18 ± 3 s; *p* < 0.05, paired Student’s *t*-test; **Table 1**; **Figure 1D**). This differential effect of activity state on inhibitory serotonergic responses in CPn and COM-biphasic neurons likely reflects the larger driving force for 1A-driven potassium conductances at depolarized potentials (Andrade and Nicoll, 1987), and activity-dependent recruitment of 2A-mediated excitation in depolarized COM-biphasic neurons. These data demonstrate that serotonergic regulation of COM and CPn neuron excitability is influenced by the level of coincident excitatory drive.

**FIGURE 2:**
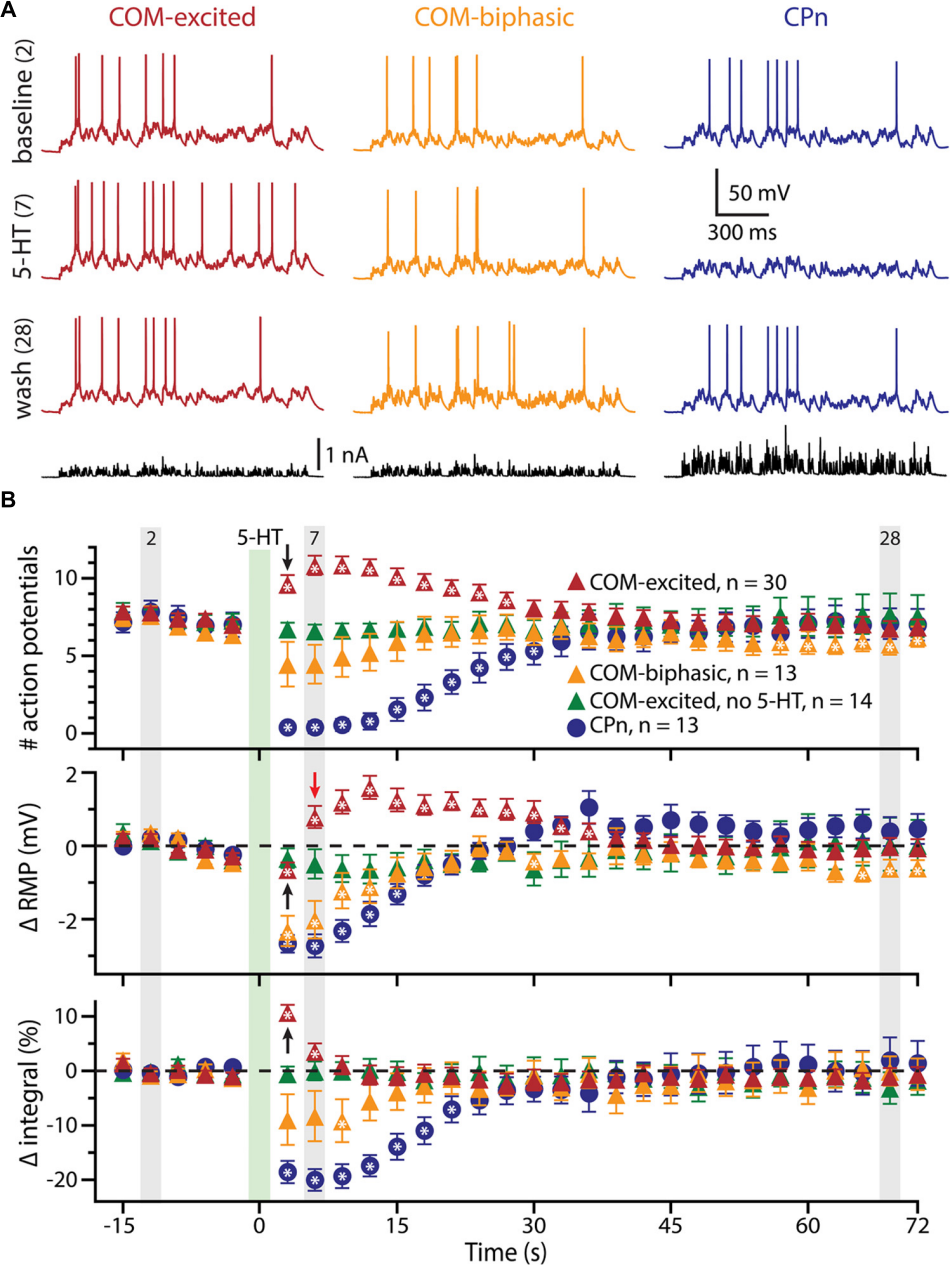
**5-HT modulates neuronal responses to simulated synaptic input. (A)** Responses of COM-excited (red), COM-biphasic (orange), and CPn (blue) L5PNs to somatic current injections simulating a barrage of excitatory synaptic input under baseline conditions (top voltage traces), after focal 5-HT application (middle voltage traces), or about one minute after 5-HT application (lower voltage traces). Injected currents (bottom traces) were scaled to generate approximately 7 action potentials (APs) in baseline conditions. **(B)** Plots of the number of APs generated by simulated synaptic input (top), changes in RMPs (middle), and percent changes in response integrals (bottom), for COM-excited (red), COM-biphasic (orange), and CPn (blue) L5PNs. 5-HT was focally applied for 1 s at the time indicated by the green bar. Gray bars indicate time-points for data shown in **A**. Green symbols indicate data from experiments in COM-excited L5PNs in which no 5-HT was applied. Asterisks indicate significant changes from baseline (*p* < 0.05). Black arrows point out COM-excited responses during trial 6 (immediately after 5-HT). Note that, while the number of APs and response integrals increase immediately during trial 6, RMP, as measured 10 ms prior to simulated synaptic input, was hyperpolarized relative to baseline in trial 6. Only following the initial post-5-HT suprathreshold current injection (trial 6) did RMPs depolarize, as observed at the beginning of trial 7 (red arrow).

To further explore the interaction of 5-HT and excitatory drive in L5PNs, we applied 5-HT to neurons receiving suprathreshold simulated synaptic input generated via somatic current injection (see Materials and Methods and **Figure 2A**). Simulating synaptic drive allowed us to deliver an equivalent excitatory stimulus to each neuron (generating ∼7 APs) while avoiding the potentially confounding presynaptic effects of 5-HT on transmitter release (e.g., Kruglikov and Rudy, 2008; Troca-Marín and Geijo-Barrientos, 2010). In CPn neurons (*n* = 13), application of 5-HT produced a 98 ± 2% decrease in the number of APs generated by the simulated synaptic current, from 7.3 ± 0.7 APs in baseline conditions to a low of 0.2 ± 0.2 APs occurring 3.5 ± 0.3 s after 5-HT application, to 7.0 ± 1.0 APs during the final trial (**Figure 2B**; blue symbols). On the other hand, 5-HT application resulted in a 59 ± 7% increase in the number of APs in COM-excited L5PNs (*n* = 30), with the peak increase occurring 8.3 ± 1 s after 5-HT application. In these neurons, 5-HT increased the number of APs from 7.5 ± 0.3 APs in baseline conditions to a peak of 11.8 ± 0.6 APs after 5-HT application, with output returning to 6.9 ± 0.5 APs on the last trial (**Figure 2B**; red symbols). Finally, 5-HT generated a transient 59 ± 12% decrease in AP number in COM-biphasic L5PNs (*n* = 13), from 7.0 ± 0.5 in baseline conditions to a low of 3.4 ± 1 APs occurring 6.5 ± 2 s after 5-HT, with recovery to 6.2 ± 0.7 APs during the final trial (**Figure 2B**; orange symbols). The maximum effects of 5-HT on AP output were significantly different among CPn, COM-excited, and COM-biphasic L5PNs (*p* < 0.05, ANOVA, **Figure 2B**).

We also monitored RMPs (as measured just prior to synaptic current injections) and the integrals of voltage responses to simulated synaptic currents (**Figure 2B**). 5-HT increased response integrals in COM-excited L5PNs, but only during the first two trials immediately after 5-HT application (trials 6 and 7; **Figure 2B**). The mean increase in response integral was 12 ± 1% over baseline (*p* < 0.05). Conversely, 5-HT induced longer-lasting decreases in response integrals in CPn neurons (mean peak change was -21 ± 2%, lasting ∼20 s; *p* < 0.05), and transient dips in response integrals in COM-biphasic L5PNs (mean peak change was -15 ± 3% relative to baseline; *p* < 0.05).

**FIGURE 3:**
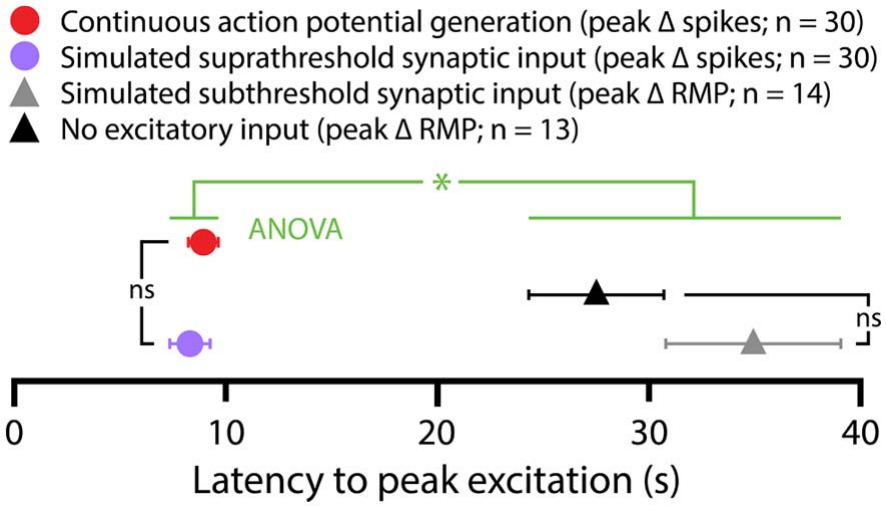
**Latencies of serotonergic responses in COM-excited L5PNs.** Plot of the latencies to peak excitatory responses (defined as peak increases in action potential frequencies or peak depolarization from RMPs) for COM-excited neurons experiencing 5-HT application during suprathreshold DC current injection (red symbol), suprathreshold (purple symbol) or subthreshold (gray symbol) simulated synaptic currents, or in the absence of any extrinsic excitatory drive (black symbol). Serotonergic responses occurring in the presence of suprathreshold drive had shorter latencies to peak than did responses at RMPs or during subthreshold simulated synaptic input (*p* < 0. 05, One-way ANOVA).

5-HT hyperpolarized CPn and COM-biphasic L5PNs by 2.9 ± 0.3 mV and 2.5 ± 0.5 mV, respectively. Hyperpolarizing responses occurred rapidly after 5-HT application, with latency to peak hyperpolarization of 4.4 ± 0.5 s in CPn neurons, and 3.7 ± 0.5 s in COM-biphasic neurons (i.e., peak hyperpolarization occurred within the first two post-5-HT trials). Conversely, 5-HT depolarized COM-excited L5PNs by 2.1 ± 0.3 mV, with peak depolarization occurring 8 ± 1 s after 5-HT application, a latency similar to the latency of peak serotonergic excitation observed in these same neurons during DC-current-induced AP generation (*p* = 0.55; paired Student’s *t*-test; see also **Figure 3**). Surprisingly, 5-HT-induced depolarization of COM-excited neurons occurred only after the initial post-5-HT exposure to simulated synaptic currents (**Figure 2B**). 3 s after 5-HT application, at the start of trial 6, but before simulated synaptic currents were applied, COM-excited neurons were *hyperpolarized* relative to baseline values (by 0.6 ± 0.2 mV; *p* < 0.05), even as AP output increased moments later in response to that same trial’s simulated synaptic input. By the beginning of the very next trial, trial 7, RMPs were significantly depolarized relative to baseline potentials (0.8 ± 0.3 mV; *p* < 0.05, **Figure 2B**), suggesting that serotonergic depolarization of COM-excited neurons may be facilitated by exogenous excitatory drive. We verified this by comparing the latency-to-peak-excitation (determined by the timing of peak increases in spike rate and/or peak depolarization of the RMP) across COM-excited L5PNs experiencing different levels of excitatory drive (**Figure 3**). Pairing 5-HT with suprathreshold DC current, or simulated synaptic currents, significantly reduced latencies to peak excitatory responses (One-way ANOVA; *p* < 0.05), with peak increases in AP generation occurring significantly earlier than peak depolarization of the RMP (Tukey-Kramer *post hoc* test, *p* < 0.05).

**FIGURE 4:**
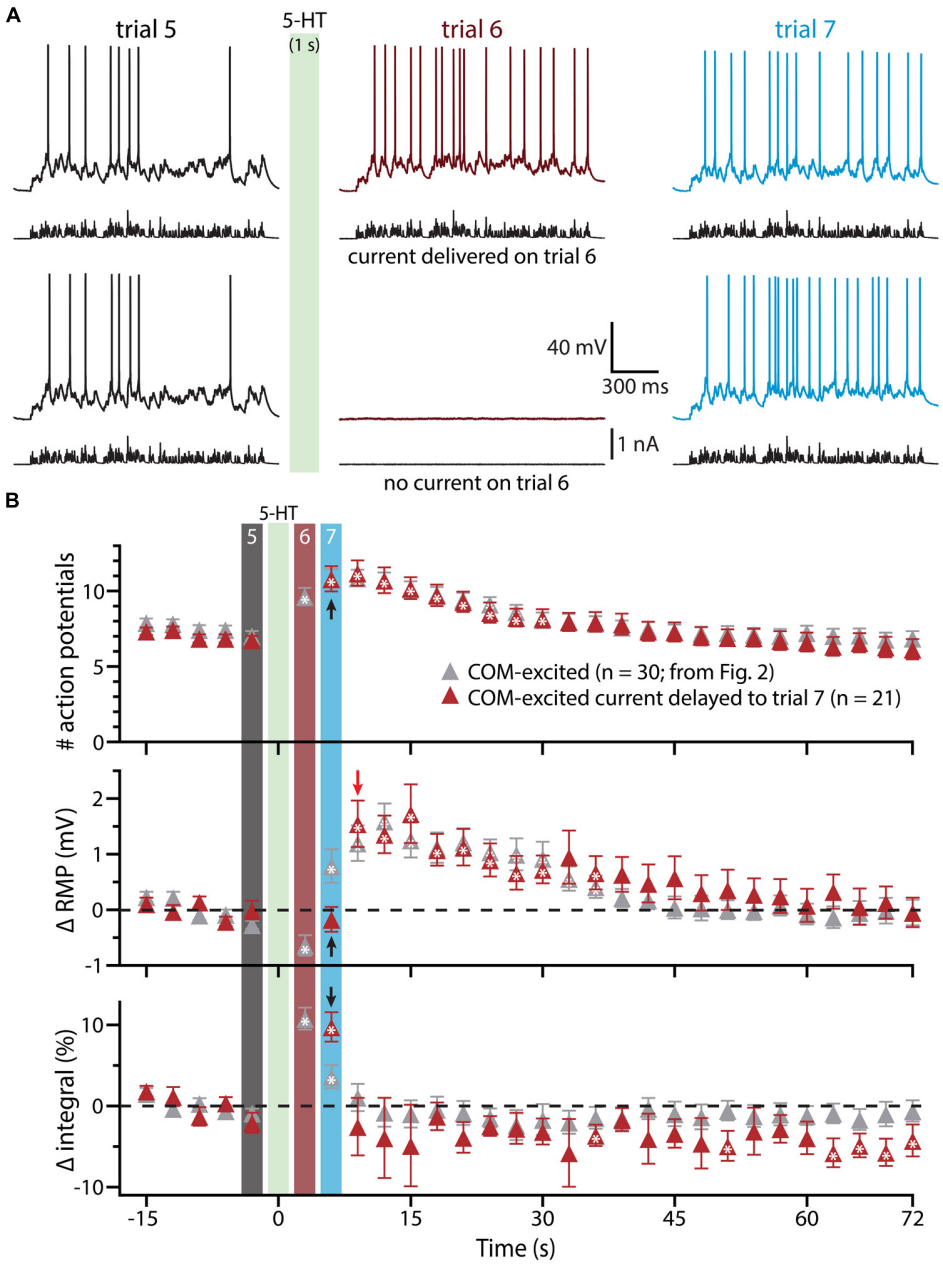
**Serotonergic depolarization of COM-excited neurons is facilitated by simulated synaptic drive. (A)** *Top traces:* responses of a COM-excited L5PN to simulated synaptic currents delivered before (trial 5; left traces), immediately following (trial 6; middle traces), or 3 s after, 5-HT application (trial 7; right traces).*Bottom traces:* responses in a COM-excited L5PN in which no simulated synaptic current was delivered during trial 6 (middle trace; “blank” trial). The green bar indicates 5-HT application between trials 5 and 6 (see panel **B**). **(B)** Plots of the number of action potentials (APs) generated by simulated synaptic current injection (top), changes in RMPs (middle), and percent changes in voltage response integrals (bottom) in COM-excited L5PNs experiencing blank trails during trial 6 (red symbols; *n* = 21). Data are superimposed on those data from COM-excited L5PNs receiving simulated synaptic current on all trials (from **Figure 2B**, gray; *n* = 30). Timing of 5-HT application shown by green bar. Responses during trials 5, 6, and 7 are indicated with black, red, and blue bars, respectively. Black arrows indicate increases in AP number and response integral, but no change in RMP, during trial 7 when neurons were not exposed to current injection during trial 6. The red arrow points out the rapid depolarization of RMPs observed at the start of trial 8. Asterisks indicate significant changes from baseline values (*p* < 0.05).

To confirm that delayed serotonergic depolarization results from the interaction of 5-HT and simulated synaptic drive, rather than reflecting a slower, time-dependent mechanism, we performed additional experiments in COM-excited neurons, in which the resumption of simulated synaptic currents was delayed by an additional 3 s (until trial 7, 6 s after 5-HT application; **Figure 4**). With this additional delay, in which simulated synaptic input was not delivered during trial 6, RMPs remained at baseline levels even at the beginning of trial 7 (6 s following 5-HT application), but depolarized sharply (by 1.5 ± 0.4 mV; *n* = 21; *p* < 0.05) after resumption of simulated synaptic input, as measured at the beginning of trial 8 (9 s after 5-HT application). These results further demonstrate that serotonergic excitation of COM-excited neurons is facilitated by extrinsic excitatory drive.

**FIGURE 5:**
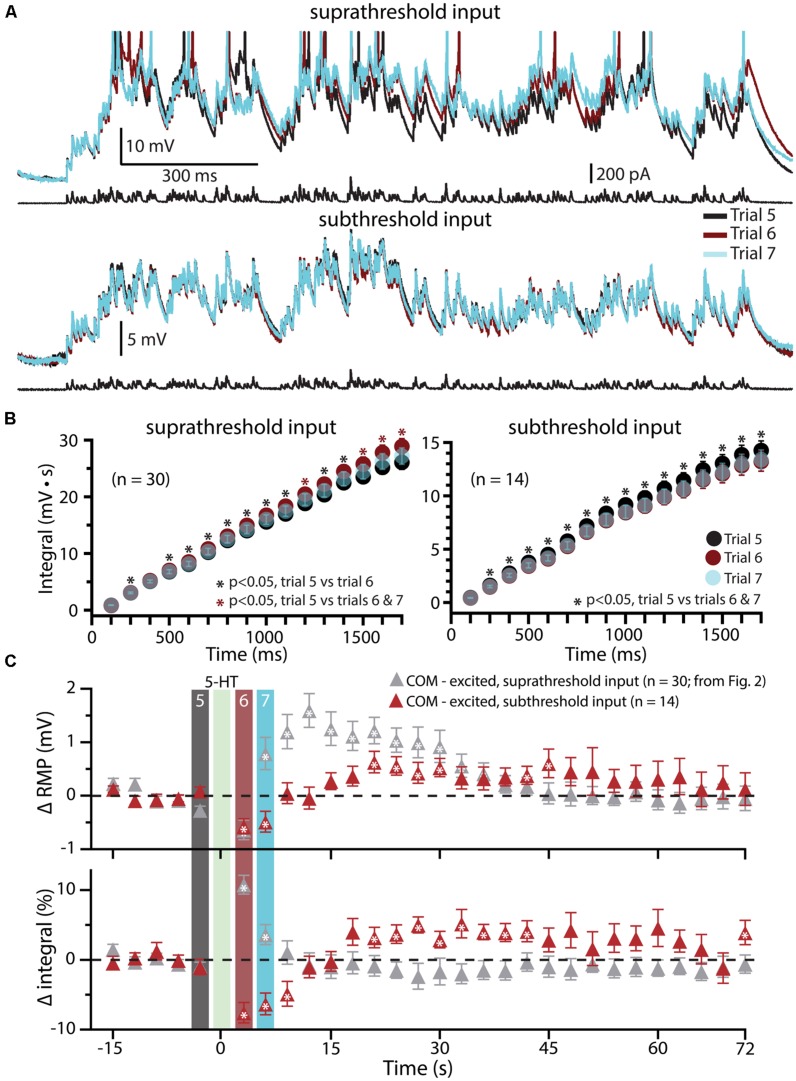
**Subthreshold simulated synaptic input does not facilitate serotonergic responses in COM-excited L5PNs. (A)** Responses of a COM-excited L5PN experiencing suprathreshold simulated synaptic input (top; action potentials truncated) or subthreshold simulated synaptic input (bottom). Baseline responses (trial 5, black) are superimposed with responses immediately after 5-HT application (trial 6, red), or 3 s later (trial 7, blue), as indicated in panel **C**. For subthreshold trials, current intensities were scaled to 80% of the minimum current necessary to elicit a single spike. **(B)** Plots of cumulative response integrals (binned at 100 ms intervals) for trials using suprathreshold (left) or subthreshold (right) simulated synaptic currents during trials 5 (black), 6 (red), and 7 (transparent blue). **(C)** Plots of changes in RMPs (top) and response integrals (bottom) for trials using subthreshold simulated synaptic input (red symbols; *n* = 14) superimposed on data from COM-excited L5PNs (from **Figure 2B**) that experienced suprathreshold simulated synaptic input (gray symbols; *n* = 30). The timing of 5-HT application is indicated by the green bar. Asterisks indicate significant changes from baseline values (*p* < 0.05).

Since previous studies in the rat PFC have found that serotonergic excitation of L5PNs is preferentially facilitated when paired with strong, rather than weak, depolarizing drive (Araneda and Andrade, 1991; Zhang and Arsenault, 2005), in a subset of COM-excited L5PNs (*n* = 14) we tested the interaction of 5-HT and subthreshold simulated synaptic drive by scaling current amplitudes to 80% of those necessary to evoke a single AP (**Figure 5**). 5-HT was focally applied after five baseline subthreshold trials, and subthreshold current injections resumed for an additional 24 trials. Under these conditions, application of 5-HT failed to promote AP generation by the simulated synaptic input (**Figure 5A**). Instead, 5-HT significantly *reduced* response integrals (by 9.2 ± 1.4%; *p* < 0.05; **Figures 5B,C**) and transiently *hyperpolarized* neurons (by 0.7 ± 0.1 mV; *p* < 0.05; **Figures 5B,C**). These results confirm that serotonergic excitation of COM-excited L5PNs is facilitated by strong, but not weak, excitatory drive.

**FIGURE 6:**
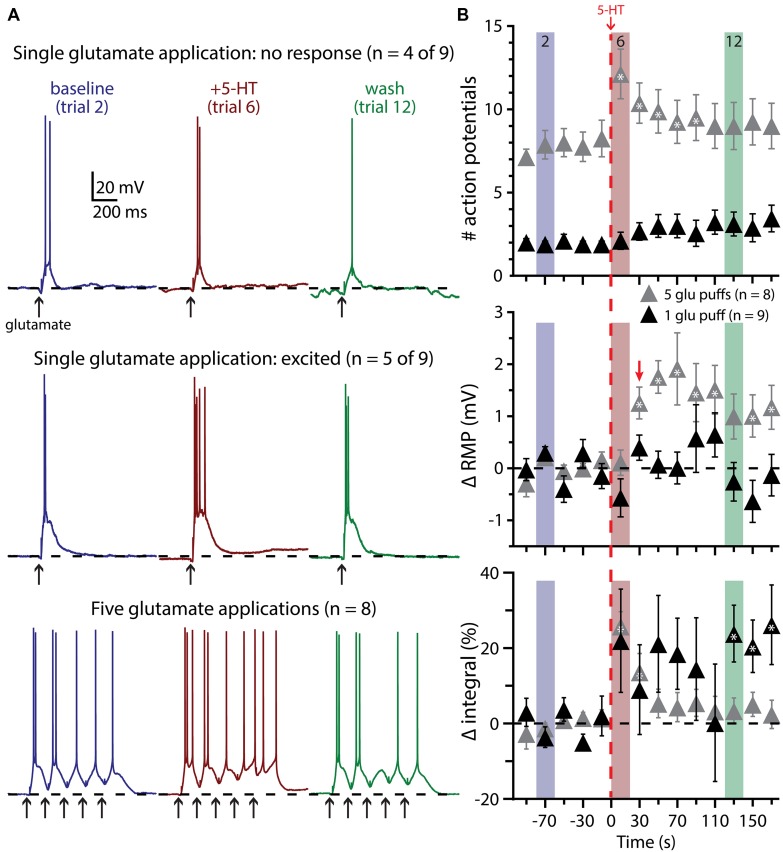
**Suprathreshold glutamatergic drive facilitates serotonergic excitation in COM-excited L5PNs. (A)** Responses of COM-excited L5PNs to single glutamate applications that were not facilitated by 5-HT application (top traces; *n* = 4 of 9) and responses of COM-excited L5PNs to single glutamate application in neurons that were facilitated by 5-HT application (middle traces; *n* = 5 of 9). Blue traces at left show baseline responses to glutamate, while red traces show responses to glutamate after 5-HT application, and green traces show responses to glutamate approximately two minutes after 5-HT exposure (“wash”). Bottom traces show responses to bursts of five glutamate applications (5 Hz) in baseline conditions (blue traces), after 5-HT application (red traces), and in wash (green traces). Dashed-lines indicates RMP. **(B)** 5-HT (red dashed-line) significantly increased the number of action potentials (APs) elicited by bursts of suprathreshold glutamate (gray symbols; top; *p* < 0.05). RMPs were significantly depolarized in neurons receiving bursts of glutamate only after the initial post-5-HT suprathreshold glutamate application (red arrow; middle; *p* < 0.05) while response integrals significantly increased immediately after 5-HT application in these neurons (bottom). 5-HT had no significant effect on AP generation or RMP in neurons receiving single suprathreshold applications of glutamate, but was associated with a slowly developing and highly variable increase in response integral. Asterisks indicate significant changes from baseline values (*p* < 0.05).

**FIGURE 7:**
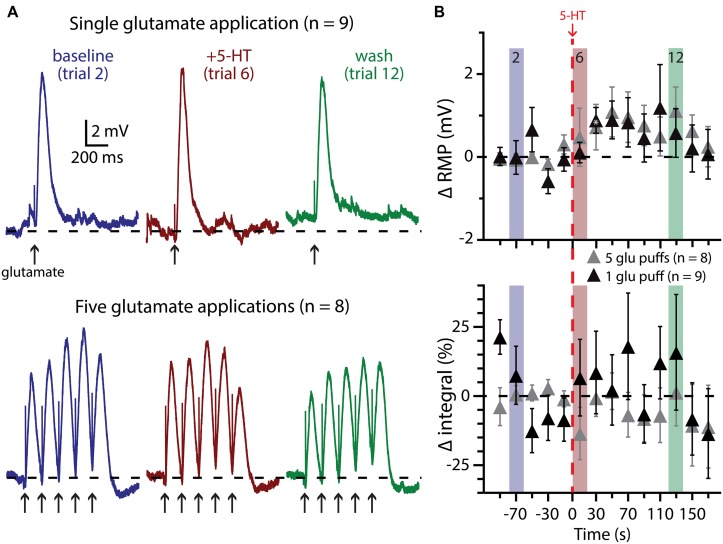
**Subthreshold glutamatergic drive fails to enhance serotonergic excitation of COM-excited L5PNs. (A)** Responses of COM-excited L5PNs to single glutamate applications (top traces; *n* = 9) or to bursts of five glutamate applications (bottom traces; *n* = 8) in baseline conditions (blue), after 5-HT application (red), and approximately two minutes later (“wash”; green). Dashed-lines indicates RMP. **(B)** Plots of changes in RMP (top) and response integral (bottom) over time for COM-excited neurons experiencing subthreshold glutamate applications. Red dashed-line indicates 5-HT application. Asterisks indicate significant changes from baseline values (*p* < 0.05).

We next tested the impact of 5-HT on the excitability of COM-excited L5PNs experiencing a second form of excitatory drive: focal application of exogenous glutamate (1 mM). In initial experiments, the duration of glutamate application (7 to 20 ms) was adjusted to generate either reliably suprathreshold responses (i.e., producing one or two APs per trial; **Figure 6**) or “just-subthreshold” responses (**Figure 7**). 5-HT (100 μM, 1 s) was applied midway between the fifth and sixth of fourteen trials delivered at 0.05 Hz. When paired with single suprathreshold applications of glutamate, 5-HT increased the number of glutamate-induced APs in about half of COM-excited neurons (*n* = 5 of 9), from 1.6 ± 0.3 APs in baseline conditions to 4.2 ± 1.1 APs following 5-HT application (**Figure 6A**). In the remaining four COM-excited neurons, 5-HT reduced the mean number of APs from 2.4 ± 0.2 in baseline conditions to 0.8 ± 0.8 APs after 5-HT application. Across all COM-excited L5PNs tested (*n* = 9), there was no significant effect of 5-HT on AP genesis or RMPs, and no immediate effect of 5-HT on response integrals, although we did observe a slowly developing and highly variable increase in response integral (**Figure 6B**; *p* < 0.05).

**FIGURE 8:**
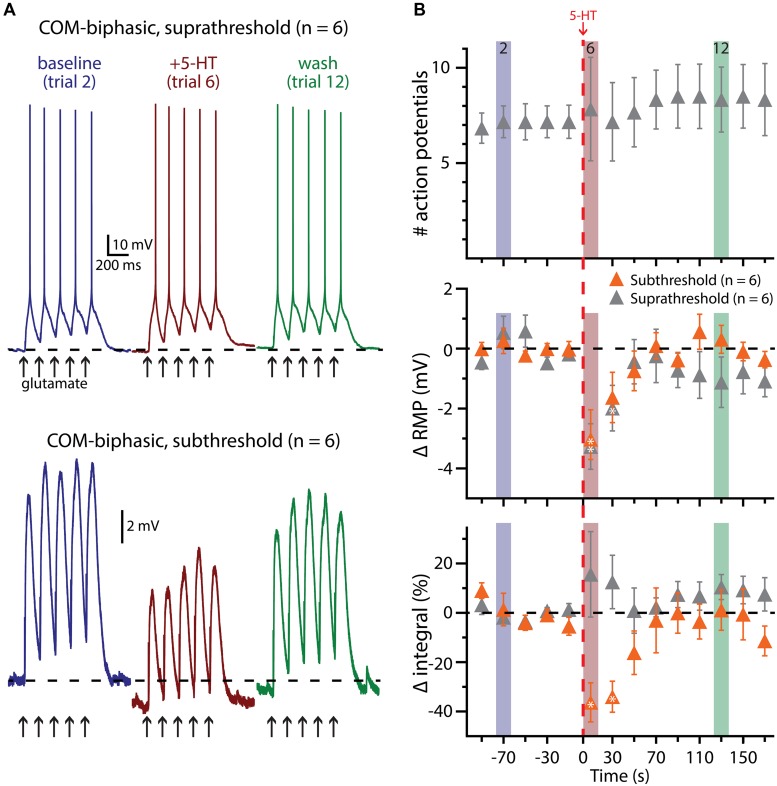
**5-HT does not facilitate glutamate-driven excitation in COM-biphasic L5PNs. (A)** Responses of a COM-biphasic L5PNs to five suprathreshold (top traces) or subthreshold (bottom traces) applications of glutamate in baseline conditions (blue), immediately after 5-HT application (red), and approximately two minutes after 5-HT application (“wash”; green). Dashed-lines indicate RMP. **(B)** Plots of the number of action potentials (APs; top), RMPs (middle), and response integrals (bottom) in COM-biphasic L5PNs receiving suprathreshold (gray symbols) or subthreshold (orange symbols) over time. 5-HT (red dashed-line) failed to enhance AP genesis, but did hyperpolarize COM-biphasic neurons, and reduced response integrals to subthreshold glutamate applications. Asterisks indicate significant changes from baseline values (*p* < 0.05).

Finally, we assessed the interaction of 5-HT and glutamatergic excitatory drive in COM-biphasic L5PNs (*n* = 6; **Figure 8**). As observed in COM-excited neurons, 5-HT failed to promote AP generation in response to subthreshold bursts of glutamate application (five applications at 5 Hz, repeated at 0.05 Hz). Instead, 5-HT hyperpolarized COM-biphasic L5PNs by 2.9 ± 0.8 mV (*p* < 0.05), and decreased response integrals by 39 ± 7% (*p* < 0.05). When paired with suprathreshold bursts of glutamate, 5-HT again failed to promote AP generation in COM-biphasic neurons (*p* = 0.8), or change response integrals (*p* = 0.4), but did induce transient hyperpolarization of 3.5 ± 0.8 mV (*p* < 0.05). The lack of obvious serotonergic inhibition in COM-biphasic L5PNs during these experiments may be the result of the long duration (∼10 s) between 5-HT application and the first post-5-HT glutamate trial, as in these neurons the inhibitory response to 5-HT during suprathreshold DC current injection lasted only 24 ± 6.6 s (*n* = 6). Together, these results confirm that serotonergic excitation of COM L5PNs is activity-dependent, promoted by suprathreshold, but not subthreshold, extrinsic excitatory drive.

These results suggest that serotonergic excitation of COM-excited neurons may be activity dependent, with the threshold for excitatory responses being multiple APs. To test whether serotonergic excitation might be enhanced by more robust glutamatergic drive, in a different group of COM-excited neurons we paired 5-HT with bursts of five glutamate applications delivered at 200 ms intervals (5 Hz), with bursts delivered at 0.05 Hz. When 5-HT was paired with suprathreshold glutamate exposure (in which APs resulted from at least three of the five glutamate applications per trial), 5-HT enhanced AP output in all neurons tested (*n* = 8). 5-HT increased glutamate-evoked output from 7.8 ± 0.8 APs in baseline conditions to a peak of 12.5 ± 1.4 APs after 5-HT application (**Figure 6B**; *p* < 0.05). 5-HT also depolarized COM-excited neurons by 2.4 ± 0.6 mV (*p* < 0.05), but only after the initial post-5-HT suprathreshold glutamate application. Finally, 5-HT increased response integrals in trials 6 and 7 by 26 ± 4% and 14 ± 5%, respectively (*n* = 8; *p* < 0.05 for each).

We also paired 5-HT with subthreshold glutamate applications, delivered individually (*n* = 9) or in bursts of five applications at 200 ms intervals (*n* = 8; **Figure 7**). In none of these experiments did 5-HT application lead to AP genesis in response to glutamate. Further, 5-HT failed to immediately enhance the integral of voltage responses to single (*p* = 0.30) or multiple (*p* = 0.22) glutamate applications. 5-HT did induce slight depolarization of the RMP during trials of single glutamate applications (in trial 7, by 0.9 ± 0.3 mV; *p* < 0.05), but not in response to bursts of glutamate, although the long (20 s) inter-trial interval limited our ability to detect subtle changes in RMP over time.

### INTERACTION OF 1A AND 2A RECEPTORS

**FIGURE 9:**
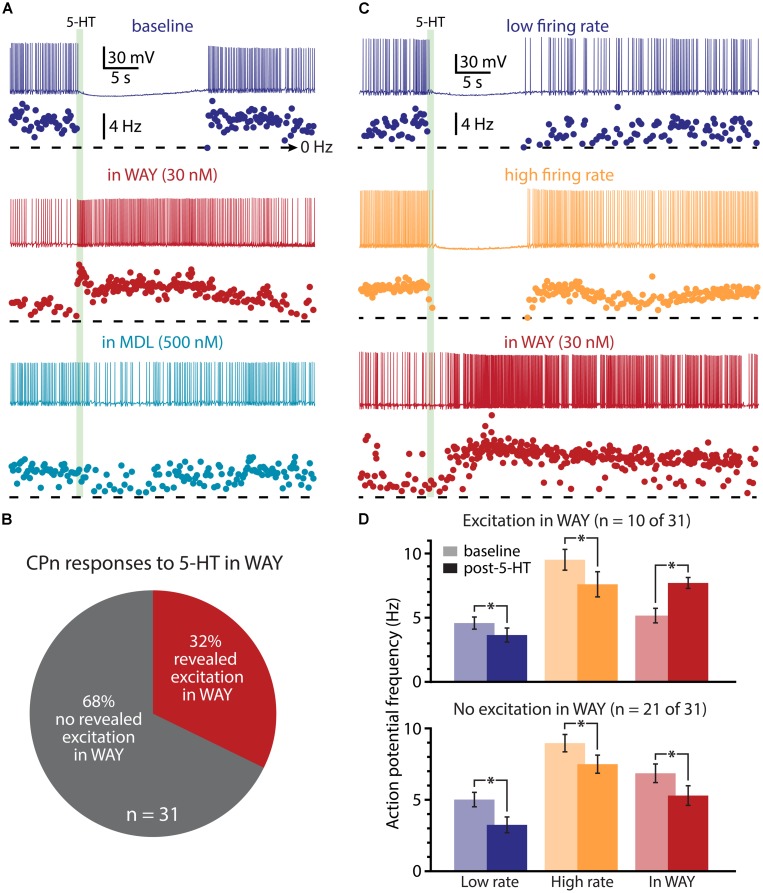
**A subpopulation of CPn neurons exhibit 2A-mediated excitation after blockade of 1A receptors. (A)** Responses of a CPn L5PN to 5-HT delivered in baseline conditions (top; dark blue), after blockade of 1A receptors by WAY 100635 (WAY; middle; red), and after additional application of the 2A antagonist MDL 11939 (MDL; bottom; light blue). Plots beneath each trace show ISF over time. The timing of 5-HT application is indicated by the green bar. **(B)** Proportions of CPn neurons exhibiting excitatory responses to 5-HT (red) or no response to 5-HT (gray) in the presence of WAY. **(C)** Responses to 5-HT (green bar) in a CPn L5PN firing at low (top trace) and high (middle trace) baseline frequency in baseline conditions, and after bath application of WAY (bottom). **(D)** Comparisons of action potential (AP) firing frequency in CPn neurons in baseline conditions (light colors) and after resumption of AP firing following 5-HT application (dark colors) for CPn neurons showing excitation in the presence of WAY (top), or no excitation in the presence of WAY (bottom). Asterisks indicate significant changes (*p* < 0.05) in post-5-HT AP frequency relative to baseline firing rates.

We next used pharmacological approaches to test the interaction of 1A and 2A receptors in generating serotonergic responses in COM and CPn neurons. As previously reported (Avesar and Gulledge, 2012), serotonergic inhibition of CPn neurons was blocked by the 1A antagonist WAY 100635 (WAY, 30 nM; **Figure 9A**; *n* = 31). In the presence of WAY, 32% of CPn neurons (*n* = 10 of 31) exhibited an excitatory response to 5-HT during DC-current-evoked AP generation (**Figure 9B**). However, the magnitude of this excitation (61 ± 14% over baseline frequencies; *n* = 10) was less robust than that observed in COM-excited L5PNs (135 ± 12% over baseline frequencies; *n* = 59; *p* < 0.05, Student’s *t*-test). Pharmacologically revealed excitation in CPn neurons was blocked by additional bath application of MDL 11939 (MDL, 500 nM; *n* = 7; **Figure 9A**), confirming the presence of functional 2A receptors in this subpopulation of CPn neurons.

Since 2A-dependent excitation is facilitated by extrinsic depolarizing drive, and given that 5-HT can increase the gain of L5PNs in the rat PFC (Araneda and Andrade, 1991; Zhang and Arsenault, 2005), we hypothesized that 2A receptors in CPn neurons might contribute to serotonergic responses when CPn neurons are driven with strong, rather than weak, excitatory drive. To test whether this was the case, we applied 5-HT to CPn neurons under two levels of excitatory drive. DC current was adjusted to generate low- (4.9 ± 0.4 Hz) or high- (9.1 ± 0.5 Hz) frequency baseline firing rates (**Figure 9C**). In both conditions, 5-HT generated hyperpolarizing responses without delayed biphasic excitation (**Figures 9C,D**). Surprisingly, *post hoc* pharmacological detection of 2A receptors was correlated with the duration of inhibitory responses only when 5-HT was paired with low-, rather than high-, frequency baseline AP generation (**Table 2**). When 5-HT was delivered during periods of low-frequency firing, the mean durations of serotonergic inhibition were 21 ± 3 s and 31 ± 3 s in “2A-responsive” and “2A-unresponsive” CPn neurons, respectively (*p* < 0.05; Student’s *t*-test). On the other hand, when 5-HT was delivered during periods of high frequency AP generation, the durations of spike inhibition were similar in 2A-responsive (17 ± 4 s) and 2A-unresponsive (20 ± 2 s) CPn neurons. In no cases did CPn firing rates increase above baseline levels following 1A-dependent inhibition. These results suggest that 2A receptor in CPn neurons can moderate serotonergic inhibition during periods of limited excitatory drive, but that under normal conditions they do not generate excitatory or biphasic responses to 5-HT.

**Table 2:** Comparison of 5-HT responses in CPn neurons with and without 2A receptors.

CPn neuron subtype	*n*	RMP (mV)	R_N_ (MΩ)	Sag (%)	Duration of inhibition (s)	
					During low frequency firing	During high frequency firing
No revealed 2A-excitation	21	-76 ± 1	82 ± 6	12 ± 1	31 ± 3	20 ± 2
Revealed 2A-excitation	10	-76 ± 1	83 ± 11	14 ± 1	21 ± 3*	17 ± 4

*Asterisk indicates *p*< 0.05 vs CPn neurons without revealed 2A responses.*

**FIGURE 10:**
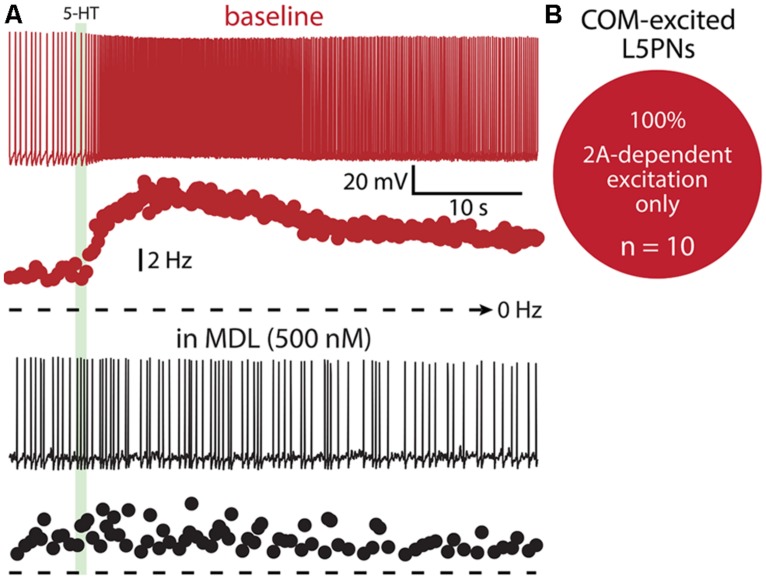
**COM-excited L5PNs do not exhibit 1A-mediated inhibition after blockade of 2A receptors. (A)** Voltage responses (top traces) and plots of ISF (lower plots) for a COM-excited L5PN exposed to 5-HT (green bar) in baseline conditions (red) and after application of the 2A receptor antagonist MDL 11939 (MDL; black). **(B)** Proportion of COM-excited L5PNs showing non-inhibitory responses to 5-HT in the presence of MDL.

Finally, we tested whether 1A receptors participate in shaping excitatory serotonergic responses in COM-excited L5PNs (**Figure 10**). When COM-excited neurons were exposed to the 2A antagonist MDL (*n* = 10), inhibitory responses were never revealed. Further, baseline firing frequencies were not correlated with the magnitude of serotonergic excitation of COM neurons (*p* = 0.22; data not shown). Thus, despite a significant proportion of COM neurons exhibiting both 1A- and 2A-receptor-mediated responses to 5-HT (i.e., COM-biphasic L5PNs), COM-excited neurons appear to respond to 5-HT solely via activation of 2A receptors.

## DISCUSSION

### ACTIVITY-DEPENDENT EXCITATION OF L5PNs

Our results demonstrate that serotonergic excitation of COM neurons is enhanced when 5-HT is paired with suprathreshold extrinsic excitatory drive. This activity-dependent facilitation of 2A-mediated excitation appears to require more than one or two APs, as 5-HT did not consistently enhance the number of APs generated by single suprathreshold applications of glutamate. Our results are consistent with findings by Araneda and Andrade (1991) and Zhang and Arsenault (2005), who, in rat L5PNs, observed that 5-HT preferentially enhanced AP generation from strong depolarizing stimuli. Our results go further, showing that in the mouse mPFC, 5-HT preferentially enhances AP output in COM-excited neurons receiving significant suprathreshold depolarizing drive, but does not boost responses to subthreshold input.

We also found that 1A-receptor-dependent serotonergic inhibition is influenced by concurrent excitatory drive, albeit to a lesser extent than is serotonergic excitation, and in opposite directions in CPn and COM-biphasic neurons. The duration of 5-HT-induced spike cessation in CPn neurons was prolonged relative to hyperpolarizing responses generated at RMPs, even in CPn neurons exhibiting 2A-dependent responses in the presence of WAY (discussed below). This effect is not unexpected, given the greater driving force at depolarized membrane potentials for the G-protein-coupled inwardly rectifying potassium channels (GIRK channels) that underly 1A-dependent inhibition (Andrade and Nicoll, 1987). On the other hand, in COM-biphasic neurons, hyperpolarizing responses generated at RMPs persisted longer than inhibition of APs during periods of suprathreshold DC current injection. This likely reflects the activity-dependent contribution of 2A receptors to serotonergic responses in these neurons; 2A-dependent excitation is expected to compete with, and limit the duration of, 1A-dependent inhibition when neurons experience suprathreshold excitatory drive.

In our experiments, we used brief (1 s) applications of exogenous 5-HT to characterize serotonergic responses in cortical neurons. During wakefulness, release of endogenous 5-HT likely generates longer-lasting stimulation of 5-HT receptors (Portas and McCarley, 1994; Sakai and Crochet, 2001). While our results suggest that COM-excited and most CPn neurons would respond to prolonged 5-HT exposure with purely excitatory and inhibitory responses, respectively, it is less clear how COM-biphasic and 2A-expressing CPn neurons might respond to tonic serotonergic stimulation. 2A receptors display a lower affinity for 5-HT, and are more prone to agonist-induced desensitization, than are 1A receptors (for review, see Zifa and Fillion, 1992), suggesting that tonic release of low concentrations of 5-HT may preferentially inhibit overall cortical output. However, 1A and 2A receptors are susceptible to heterologous desensitization (Zhang et al., 2001; Carrasco et al., 2007), raising the possibility of complex interaction among 1A and 2A signaling *in vivo*. Additional studies will be necessary to explore the response of COM and CPn neurons to tonic exposure to physiological concentrations of 5-HT, and to endogenous release of 5-HT *in vivo*.

### INTERACTION OF 5-HT RECEPTORS

The antagonistic interplay of 1A and 2A receptors in regulating behavior is well established (Berendsen and Broekkamp, 1990; Darmani et al., 1990; Willins and Meltzer, 1997; Carli et al., 2006), and direct interaction of 1A and 2A receptors within individual cortical pyramidal neurons has long been hypothesized (Ashby et al., 1994; Martín-Ruiz et al., 2001; Amargós-Bosch et al., 2004). Yet, while many L5PNs in the rodent PFC express both 1A and 2A receptors (Martín-Ruiz et al., 2001; Santana et al., 2004; Wȩdzony et al., 2008; Vázquez-Borsetti et al., 2009), most respond to 5-HT with unidirectional, 1A- or 2A-mediated, responses (Araneda and Andrade, 1991; Tanaka and North, 1993; Spain, 1994; Zhang, 2003; Benekareddy et al., 2010; Avesar and Gulledge, 2012), with 1A-mediated inhibition predominating in the mature cortex (Béïque et al., 2004; Puig et al., 2004, 2005). The prevalence of 1A-mediated inhibition may reflect the greater abundance of CPn L5PNs relative to COM L5PNs (Hattox and Nelson, 2007), and/or the activity-dependence of 2A-mediated excitation, as pyramidal neurons are generally quiescent *in vitro* and have reduced excitatory drive under anesthesia *in vivo* (Hentschke et al., 2005).

Our results also confirm that a significant proportion of CPn neurons (∼32%) express functional 2A receptors capable of generating modest excitatory responses when 1A receptors are pharmacologically blocked. This proportion is comparable to the proportion of rat prelimbic neurons found to coexpress 1A and 2A mRNA (∼41%; Santana et al., 2004) or protein (∼38%; Wȩdzony et al., 2008), and is similar to the proportion of COM neurons exhibiting 1A-mediated biphasic inhibition (∼35%; Avesar and Gulledge, 2012). Yet, even when present, 2A receptors have only limited impact in shaping serotonergic responses in CPn neurons. We found the presence of 2A receptors to influence the duration of inhibitory responses when 5-HT was delivered during periods of low, but not high, frequency AP generation. This contrasts with the activity-dependence of serotonergic excitation in COM neurons, and with the hypothesis that 2A receptors generally increase the gain of cortical pyramidal neuron output (Araneda and Andrade, 1991; Zhang and Arsenault, 2005). While more studies will be necessary to evaluate the role of 2A receptors in regulating excitability in CPn neurons, it is also possible that 2A expression primarily serves alternative functions, such as regulation of synaptic transmission and/or dendritic excitability in ways not readily observable in our experiments (Carr et al., 2002; Yuen et al., 2008; Zhong et al., 2008; Troca-Marín and Geijo-Barrientos, 2010).

One cortical circuit influenced by both 1A and 2A receptors provides positive feedback to serotonergic neurons in the dorsal raphe; injection of the selective 2A agonist DOI into the mPFC increases the local release of 5-HT (Martín-Ruiz et al., 2001; Puig et al., 2003). Since 2A receptors are expressed in subpopulations of brainstem projection neurons (Martín-Ruiz et al., 2001; Santana et al., 2004), one possibility is that DOI directly excites cortico-raphe neurons. However, 2A-dependent increases in 5-HT release required, and were mimicked by, intracortical glutamatergic transmission (Martín-Ruiz et al., 2001; Puig et al., 2003), suggesting a role for indirect excitation of cortico-raphe neurons from the directionally selective synaptic connectivity between 2A-expressing COM L5PNs and brainstem-projecting neurons (Morishima and Kawaguchi, 2006). Martín-Ruiz et al. (2001) also demonstrated that 1A receptor activation can suppress the effects of DOI, confirming an antagonist relationship between 1A and 2A receptors in regulating cortical circuits, and the primacy of 1A receptors in regulating the overall output of L5PNs projecting to the brainstem.

### MECHANISMS OF SEROTONERGIC REGULATION OF L5PNs

Although there is general consensus that 1A-dependent inhibition of cortical pyramidal neurons is mediated by G_i/o_-coupled GIRK channels (Andrade et al., 1986; Lüscher et al., 1997), the mechanisms responsible for 2A-dependent excitation of cortical neurons remain uncertain. We previously observed that 2A-dependent excitation remains intact in the presence of blockers of fast synaptic transmission (Avesar and Gulledge, 2012). Similarly, 5-HT can enhance AP generation resulting from current injection or exogenous glutamate, suggesting that 5-HT has direct effects on the intrinsic excitability of COM L5PNs (see also Béïque et al., 2007). Yet, the ionic mechanisms responsible for intrinsic 2A-dependent excitation remain mysterious. One possibility is that 2A receptor activation suppresses potassium conductances facilitated by depolarization, including “M-like” currents (McCormick and Williamson, 1989; Tanaka and North, 1993; Zhang, 2003) and the calcium-dependent potassium conductances associated with slow afterhyperpolarizations (Araneda and Andrade, 1991; Pedarzani and Storm, 1993; Villalobos et al., 2005, 2011), although this later effect appears to be limited at physiological temperatures (Spain, 1994; see also Gulledge et al., 2013). Another attractive possibility is that 2A receptors enhance voltage-sensitive cationic conductances similar to, or perhaps identical to, those mediating cholinergic excitation in prefrontal L5PNs (Haj-Dahmane and Andrade, 1996, 1999; Yan et al., 2009; but see Dasari et al., 2013). Less likely are direct actions of 5-HT on voltage-gated sodium or calcium channels, as 2A receptors are generally considered negative regulators of these conductances (Bayliss et al., 1995; Carr et al., 2002). While the ionic effectors mediating serotonergic excitation remain unknown, the ability to selectively target 2A-excited L5PNs (i.e., COM L5PNs) in the mouse mPFC may facilitate future studies focusing on 2A-dependent postsynaptic signal transduction.

### FUNCTIONAL IMPLICATIONS OF ACTIVITY-DEPENDENT SEROTONERGIC EXCITATION OF COM NEURONS

Serotonergic input to the PFC facilitates behavioral inhibition, and depletion of prefrontal 5-HT produces impulsive behaviors in both animals and humans (Harrison et al., 1997; Winstanley et al., 2004b; Worbe et al., 2014). Studies in rodents have dissociated the roles of 1A and 2A receptors in regulating impulsive behavior, finding that 2A receptor agonists enhance (Koskinen et al., 2000; Winstanley et al., 2003; Blokland et al., 2005), while 1A agonists and 2A antagonists suppress (Higgins et al., 2003; Winstanley et al., 2003; Carli et al., 2004, 2006; Blokland et al., 2005; Fletcher et al., 2007) impulsivity. Thus, the effect of 5-HT on cortical circuits will depend, in part, on the net balance of 2A-dependent excitation of COM-excited neurons and 1A-dependent inhibition of COM-biphasic and CPn neurons. Our results suggest that pharmacological enhancement of 2A-dependent excitation, without enhanced 1A-dependent inhibition, may contribute to impulsivity via non-specific amplification of intracortical networks. On the other hand, blockade of 2A receptors, or activation of 1A receptors, is expected to reduce overall cortical drive. Given that most L5PNs in the adult neocortex exhibit 1A-mediated inhibition (Béïque et al., 2004; Avesar and Gulledge, 2012), and that 5-HT directly excites subpopulations of GABAergic interneurons via ionotropic 5-HT_3_ receptors (Morales and Bloom, 1997; Puig et al., 2004; Lee et al., 2010), increased cortical 5-HT might be expected to reduce impulsivity by limiting output from the PFC. This may well be the case, as serotonergic tone in the mPFC is negatively correlated with impulsivity (Barbelivien et al., 2008), and selective 5-HT reuptake inhibitors (SSRIs) that boost 5-HT levels in the mPFC (Jordan et al., 1994) generally reduce impulsivity (Baarendse and Vanderschuren, 2012). The activity-dependent serotonergic excitation of COM L5PNs described here may further help reduce impulsivity by restricting 2A-dependent amplification of cortical output to behaviorally relevant circuits.

## Conflict of Interest Statement

The authors declare that the research was conducted in the absence of any commercial or financial relationships that could be construed as a potential conflict of interest.
